# Implementing the EU HTA regulation and joint clinical assessment: a multi-stakeholder perspective from Italy

**DOI:** 10.1017/S026646232610364X

**Published:** 2026-04-13

**Authors:** Michela Meregaglia, Francesco Costa, Ludovico Cavallaro, Patrizio Armeni, Monica Hildegard Otto

**Affiliations:** SDA Bocconi School of Management, Italy

**Keywords:** Health Technology Assessment (HTA), HTA Regulation (HTAR), Joint Clinical Assessment (JCA), stakeholders, Italy

## Abstract

**Background:**

The Regulation (EU) 2021/2282 on Health Technology Assessment (HTAR), which applies as of January 2025, introduces the Joint Clinical Assessment (JCA) for selected health technologies and establishes a stakeholder network. This study aims to evaluate the expected impact of the implementation of the HTAR from a multi-stakeholder perspective, using Italy as a case study.

**Methods:**

A scoping literature review was performed according to the PRISMA guidelines to inform the development of an interview guide. Target participants included Italian stakeholder representatives with an interest in the HTAR. One-on-one semi-structured interviews were conducted virtually at the end of 2024. The questions were categorized into three main topics: expected benefits and opportunities; foreseen risks or challenges; recommended actions. The interviews were transcribed verbatim and analyzed using thematic content analysis techniques.

**Results:**

Thirteen participants included representatives from national and regional HTA bodies, health technology developers’ associations, health professional associations, patient organizations, and HTA experts. The JCA is expected to enhance the quality of clinical assessment and to result in faster and more equitable access to health innovations. However, the timing will depend on the extent to which Member States require complementary analyses. Health technology developers benefit from submitting a single JCA dossier, but often cope with limited evidence and short-term deadlines. The interviewees recommended harmonizing evidence standards, investing in HTA education and training, and fostering strategic stakeholder collaborations.

**Discussion:**

The process of harmonization induced by the HTAR is beneficial to standardize clinical assessment at the EU level, but needs to reconcile different stakeholder perspectives.

## Background

In December 2021, the Regulation (EU) 2021/2282 on Health Technology Assessment (HTAR) was finally adopted by the European Parliament and the Council of the European Union. The HTAR came into force in January 2022 and applies as of January 2025 (1;2). The Regulation was designed to formalize and make mandatory and sustainable in the long-term some of the initiatives that had been pursued over several years on a voluntary basis, through joint actions such as the European Network for Health Technology Assessment (EUnetHTA). It aimed at fostering formal, systematic, and transparent collaboration and reconciling divergent approaches among national HTA agencies through the establishment of standardized methodologies and procedures (2). So far, after receiving marketing authorization from the European Medicines Agency (EMA) or national regulatory bodies, manufacturers must file applications with HTA agencies of individual Member States (MS) for pricing & reimbursement (P&R), which, however, have heterogeneous evidentiary standards, assessment criteria, procedures, and timelines (3). The HTAR promotes efficient use of resources dedicated to HTA by reducing duplication of efforts for national HTA authorities and health technology developers (HTDs), and provides the legal basis for permanent and sustainable cooperation between national HTA bodies at the EU level (4). By harmonizing HTA processes across countries, the HTAR allows them to reconcile inter-country tensions related to different demographic, epidemiological, and economic situations, and avoid discrepancies in access to health technologies across Europe (5). Moreover, it emphasizes a reliance on clinical evidence and ensures a uniform decision-making basis for clinical assessment at the EU level, thus ultimately favoring value-based pricing decisions by individual MS.

In terms of governance, the HTAR foresees the establishment of a Coordination Group (HTACG), composed of representatives of the MS, and the creation of subgroups to perform technical HTA work. Under the HTAR, for the first time, the clinical assessment of health technologies is centralized at the EU level through a joint clinical assessment (JCA) and conducted in parallel with the EMA procedure for obtaining marketing authorization. The Regulation, indeed, applies to the clinical domains of HTA, that is, the relative effectiveness and the relative safety of the new technologies compared to the existing ones (6). The first technologies subjected to JCA starting in January 2025 were anti-cancer drugs and advanced therapy medicinal products (ATMPs), which will be followed by orphan medicinal products in 2028. From 2030 onwards, the JCA will be extended to all other centrally approved medicines. In 2026, the first JCAs for medical devices are planned to be initiated, with approximately five JCAs covering a selection of devices that include in vitro diagnostics (7). The HTAR establishes a framework for Joint Scientific Consultations (JSCs) at the EU level to advise HTDs (i.e., pharmaceutical and device manufacturers) on clinical study designs for generating the appropriate evidence that is required for a future JCA (8). The JCA involves a harmonized assessment process where individual countries collaborate to evaluate the clinical evidence and value of health technologies based on a pre-agreed PICO (population, intervention, comparator, outcome) framework across MS. The PICO survey, a key component of the JCA scoping process, allows MS to express their specific needs and preferences regarding the PICO elements for a given technology. This can lead to potential multiple PICOs for a single technology, especially when the standard of care is not well-defined, such as in rare diseases. Based on the consolidation of PICO survey results by the JCA subgroup, which defines the data requirements, the HTDs prepare a single JCA dossier and submit a single EU-wide application with clinical evidence for JCAs, rather than performing multiple submissions in parallel to different national HTA agencies. The JCA dossier needs to acknowledge all PICOs identified through scoping and be submitted within around 100 days from the end of the PICO survey (4). In carrying out HTA and decision-making at the national level, MS shall give due consideration to the results of a JCA but may supplement them with additional clinical data, analyses, and information required by their national HTA procedures or health situation (9), such as epidemiological trends and healthcare system structures. The centralization of comparative clinical assessment is expected to allow MS to focus on non-clinical (i.e., economic, ethical, organizational, social, environmental, and legal) domains of HTA, which remain the responsibility of the national bodies (10;11). The MS, however, can engage in voluntary cooperation with other MS in these areas, and continue to be responsible for drawing conclusions on the overall added value of health technology and related P&R decisions. The EU framework puts more emphasis on the involvement and participation of relevant stakeholders in the joint HTA. For example, the European Access Academy (EAA), founded in 2021, is a multi-stakeholder initiative that supports the HTAR vision of increasing patient access to life-saving innovative medicines. During the 2022 Fall Convention, four key stakeholder groups were identified: patients and patients’ representatives; clinicians, healthcare practitioners, and medical societies; regulators; industry associations and HTDs (12).

The HTAR expressly provides for the establishment of a Stakeholder Network (SN) to support the implementation of the Regulation itself. The first SN was created in 2023 with 44 European organizations, including patient associations (31 percent), health professional organizations (27 percent), HTD associations (16 percent), healthcare payer organizations (7 percent), scientific associations (7 percent), and others (13 percent), plus two organizations as observers (13;14). In September 2024, the European Commission (EC) launched a supplementary call for applications with the purpose of selecting additional organizations to the existing network before the date of application of the Regulation (1;14).

In Italy, the Italian Medicines Agency (AIFA) is responsible for granting marketing authorization as well as for defining the eligibility for reimbursement of the authorized medicines and negotiating their price with HTDs. Italy, with AIFA, actively participated in preparatory activities for the HTAR and faced the challenge of adapting its national process simultaneously to the EU rules and to AIFA’s reform, which started in November 2022 to make its work more efficient and to respond to the growing complexities of the health technology sector (3). In place of two separate Committees dealing with, respectively, defining medicines’ therapeutic value and innovativeness, and decisions on P&R, a single Scientific and Economic Committee was introduced to shorten the approval time of dossiers and speed up patient access to medicines (15). AIFA is currently a member of the HTACG and of all the subgroups in the medicinal products configuration (16). A member of AIFA was also elected as co-chair of the Emerging Health Technologies Subgroup (17).

This study aims to identify the expected benefits and risks deriving from the implementation of the HTAR at the national level from a multi-stakeholder perspective, and provide recommendations to facilitate this process, using Italy to perform an in-depth case study.

## Methods

We performed a scoping literature review followed by semi-structured stakeholder interviews. The literature review was performed to gain a broad European perspective on the topic and to shape the questions of the interview guide. The subsequent interviews allowed us to gain insights into the Italian case study and to contextualize literature findings from other settings.

### Literature review

A scoping literature review covering legislative, peer-reviewed, and grey literature was performed according to PRISMA for Scoping Reviews (PRISMA-ScR) (18). The scientific literature was searched in PubMed by title/abstract from January 2022 (when the Regulation entered into force) until 9th December 2024. The search strategy involved a combination of keywords, including “EU OR European OR 2021/2282,” “regulation,” and “Health Technology Assessment OR HTA.” The retrieved articles were included if in English or Italian and addressed the expected impacts of the HTAR. We also examined the full-text of the HTAR (1) and the contents of institutional websites of organizations (i.e., EC, EUnetHTA, EMA, and AIFA) involved in HTA to complement and deepen the information retrieved from the scientific literature.

### Semi-structured interviews

A semi-structured interview topic guide was developed based on the findings of the literature review and included a list of standardized open questions (Supplementary Material S1). The questions were categorized into three main topics: (1) expected benefits and opportunities arising from the HTAR and JCA implementation; (2) expected risks or challenges; (3) actions that may be required at the national or international level to comply with the HTAR procedures. The questions were broad enough to allow participants to argue on other topics related to the HTAR. Target participants included Italian representatives of organizations and associations having an interest in the implementation of the HTAR (i.e., national and regional HTA bodies, HTD associations, health professionals’ organizations, patient associations, and HTA experts). The purpose was to recruit at least two from each stakeholder group, without replicating their distribution within the SN. The relevant stakeholders were preidentified using purposive sampling, based on their role, knowledge, and experience. Invitations were sent via email together with the informed consent form, a short study description, and the interview topic guide. One-on-one, semi-structured in-depth interviews were conducted and recorded via teleconference (Teams) after obtaining the participant’s consent. In replying to each question, participants were invited to embrace mainly their stakeholder group’s perspective. At the end of the interview, respondents could refer to other potential participants through snowball sampling. The participants were not financially compensated for their time. The audio recordings were transcribed verbatim, pseudonymized (i.e., every participant received a unique identifier based on their stakeholder group) to protect participants’ confidentiality, and analyzed using thematic content analysis techniques. The interview transcripts were read, reread, and organized based on key topics according to the topic guide and the study objectives. The study received approval from the Bocconi Ethics Committee on November 19th, 2024 (study code: EA000842).

## Results

### Scoping literature review

**Figure 1.**
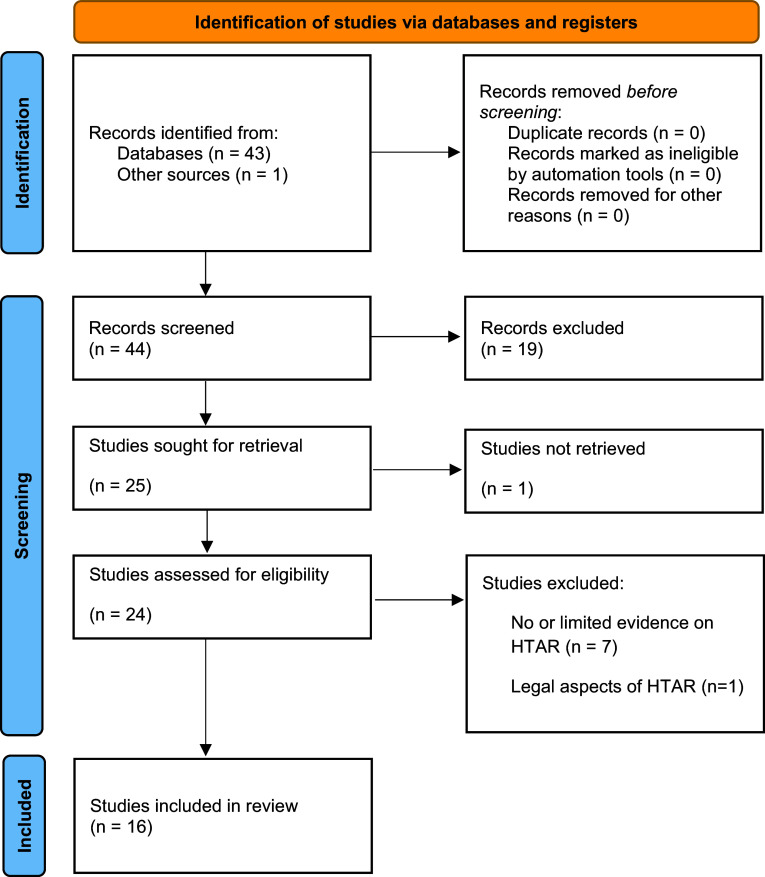
PRISMA flow diagram.

A total of 44 unique records were retrieved, of which 43 were from PubMed, and one was obtained using a snowballing approach. Screening on title and abstract excluded another 19 articles, leaving 25 articles for full-text review. Of these, one was not retrieved, and eight were excluded because the topic of the HTAR was only marginally addressed. Therefore, 16 articles (4–6;19–31) were finally included (Figure 1). A synthesis of the included studies is reported in Table 1. The majority (13 of 16) adopted a multi-country perspective, while the remaining three were focused on individual MS (i.e., two on Belgium and one on Malta). Ten studies embraced the perspective of all stakeholder types included in the SN or did not specify the stakeholder type; two were focused on HTDs, one on clinician and patient experts, and the remaining three on different groups of stakeholders. The specific topics addressed by the included studies were heterogeneous, and encompassed HTA capacity building, countries’ readiness for the implementation of the HTAR, impact on national HTA procedures, stakeholders’ involvement in the SN and related issues, and assessment of specific products (e.g., cancer medicines and medical devices). The studies identified different challenges for MS, including their readiness for JCAs, capacity and capability constraints, adjustability of national laws and health policy processes, methodological uncertainties, and differences in evidence standards. Therefore, several actions are indicated to MS in order to facilitate their transition toward the implementation of the HTAR, such as capacity building activities at individual and organizational level, developing HTA guidelines as more flexible (“living”) documents, developing a clear PICO identification process, revising national timelines and processes to comply with the JCA and JSC, investing in training and education, and preparing for interactions at European level. Regarding the SN, engaging in an “inclusive civil society dialogue,” ensuring expert diversity, as well as properly managing conflicts of interest, are key factors to ensure a balanced, impartial, and transparent stakeholders’ involvement in the network.

**Table 1.** Synthesis of included studies (n = 16)

First author, Year	Country	Study design	Specific topic	Stakeholder type(s)	Key findings/conclusions
Abraham, 2024	Malta	Literature review; semi-structured interviews (n = 33)	HTA capacity building	Policy makers, appraisal committee members, procurement staff, medical specialists, pharmacists, and pharmaceutical industry representatives.	HTA capacity building activities should address the individual level (i.e., skill training on the ability to perform HTA), organizational level (i.e., development of a new HTA framework to make the use of HTA’s more effective and efficient), and the environmental level (i.e., awareness campaign).
Brinkhuis, 2024	Any	Four break-out sessions within the 2023 EAA Convention	Readiness and remaining challenges for the HTAR implementation	All (or not specified)	Overall, “readiness” was perceived as neutral. Prioritized action points identified in the four sessions included the following: (i) health policy, that is assess adjustability of MS laws and health policy processes; (ii) stakeholders, that is capacity building; (iii) uncertainty, that is implement HTA guidelines as living documents; (iv) methodology, that is clarify the PICO identification process.
Brinkhuis, 2024	15 countries	Multi-stakeholder semi-quantitative survey (61 responses)	Stakeholders’ perceptions of the progress made towards, and remaining challenges for, the HTAR implementation	Patients and patients’ representatives; clinicians’ representatives; regulators; HTDs; HTA bodies; payers; policy makers; academic representatives.	Respondents ranked the success of preparatory activities neutral with a slight tendency towards a positive ranking. Key challenges were MSs’ readiness for JCAs, HTA capacity/ capability constraints, and the applicability/feasibility of the methodological framework.
Desmet, 2024	Belgium, any	Scoping literature review; semi-structured interviews (n = 20)	Impact of the regulation on national HTAs, the strategic implications for HTDs, and its influence on P&R negotiations	Belgian and European institutions; patients; ATMPs developers; academics; sickness funds	At MS level, national/regional HTA bodies and payers must act to adopt the new concepts of JSC and JCA within their national legislation, as well as revise their timelines and prepare for interactions at a European level. Training and education will help HTA bodies, payers, and HTDs to participate in the European processes.
Desmet, 2024	Any	Working group sessions within the 2022 EEA Convention	Involvement of Stakeholders and Collaborators	All (or not specified)	Addressing an “inclusive civil society dialogue” starts with the implementation of strong and balanced stakeholder involvement in the EU HTA process.
Drummond, 2022	Any	Opinion	Issues that need to be addressed for the HTAR to succeed	All (or not specified)	The new HTAR represents an important milestone that is expected to change the HTA landscape in Europe, but some critical issues should be tackled: differences in current standard-of-care; lack of randomized clinical studies; use of surrogate endpoints; and real-world data generation.
Gentilini, 2024	Any	Review	Conflicts of interest in the JCA	Individual experts (i.e., clinicians and patient representatives)	Improvements in transparency, expert diversity, and comprehensive COIs management are needed to ensure impartiality in the JCA process.
Gsteiger, 2024	Any	Opinion	Assessment versus appraisal	All (or not specified)	Reimbursement decision-making should be structured into two stages: a first scientific step (the assessment), followed by a categorization of the overall added benefit in a local context (the appraisal).
Hulstaert, 2023	Belgium	Review of assessor reports and literature	Level of evidence for innovative high-risk medical devices at market entry	All (or not specified)	The HTAR plans for JSC for specific high-risk devices before companies begin their pivotal clinical investigations, as well as a JCA that feeds into the HTA at a national or regional level.
Hwang, 2022	Any	Opinion	Implications of HTAR for cancer medicines	All (or not specified)	Since cancer medicines are frequently authorized on the basis of surrogate endpoints, joint HTA reviewers will probably need to make indirect comparisons.
Julian, 2022	Any	Preparatory multi-stakeholder survey; working groups at the EAA inaugural convention 2022	Research agenda to ensure a successful HTAR implementation	All (or not specified)	19 domains were identified in relation to the four key areas (processes, uncertainty, comparator choice, and endpoint selection) that urgently need to be addressed for the HTAR to become a success.
Karres, 2024	Any	Review	Evidence generation for pediatric medicines	All (or not specified)	Early interactions with all relevant stakeholders, particularly between regulators and HTA bodies, are key to optimizing data generation and its utility for pediatric populations as facilitated by collaborations through JSC.
Largeron, 2024	Any	Literature reviews, expert consultation	Guiding principles for JCA of vaccines	All (or not specified)	13 recommendations were developed to help inform the development of standardized and vaccine-specific methodologies and processes for use in the EU joint HTA framework.
Reason, 2024	Any	Proof-of-concept	Mass extraction of PICOs from abstracts using GenAI	HTDs	Using GenAI to extract PICOs from clinical study abstracts enables HTDs to anticipate PICO requirements and proactively prepare for the HTAR process.
Schuster, 2024	Any	Opinion	EU HTA regulation and analyze threats and opportunities for manufacturers	HTDs	Industry experts see the risk of additional bureaucracy resulting in delays, further scrutiny, and one additional EU (clinical) dossier to submit on top of all national HTA dossiers.
Tarricone, 2023	Any	Two international workshops and one consensus group conference	Access pathway for innovative high-risk medical devices under the new EU MDR and HTAR	All (or not specified)	The new EU MDR and HTAR together will hopefully increase the quality of clinical evidence for MDs and reduce fragmentation in the market access process in the EU; however, they may also result in delays for certain types of innovative, high-risk MDs.

ATMPs: advanced therapy medicinal products; COI: conflict of interest; EAA: European Access Academy; HTA: health technology assessment; HTAR: health technology assessment regulation; HTD: health technology developer; JCA: joint clinical assessment; JSC: joint scientific consultations; MD: medical device; MDR: medical device regulation; MS: Member State; P&R: pricing and reimbursement; PICO: population, intervention, comparator, outcome.

### Sample’s characteristics

Of the 13 participants invited to participate in this study, none declined participation. The recruited participants represented all potential stakeholders involved in the HTA of medicinal products at different institutional levels in Italy. They were interviewed for about half an hour between mid-November and mid-December 2024 (Supplementary Material S2). The composition of the participants included one representative (N1) from the Italian HTA authority, two representatives (R1 and R2) from regional HTA bodies, three industry representatives (D1, D2 and D3), two clinician group representatives (C1 and C2), three patient association and education representatives (P1, P2 and P3), and two HTA experts (E1 and E2). The latter were selected based on their in-depth knowledge of HTA at different levels. The majority were female (61.5 percent) and practicing their profession at the national level (61.5 percent), as shown in Table 2.

**Table 2.** Participants’ characteristics (n = 13)

	N	%
**Organization type**		
HTA bodies:	3	23
*- At national level (AIFA)*	*1*	*7.6*
*- At regional level*	*2*	*15.4*
Health technology developer	3	23.1
Health professional	2	15.4
Patient association/education	3	23.1
Expert	2	15.4
**Scope of work**		
International	2	15.4
National	8	61.5
Regional	3	23.1
**Gender**		
Female	8	61.5
Male	5	38.5

AIFA, Italian Medicines Agency.

### Summary of themes

**Figure 2.**
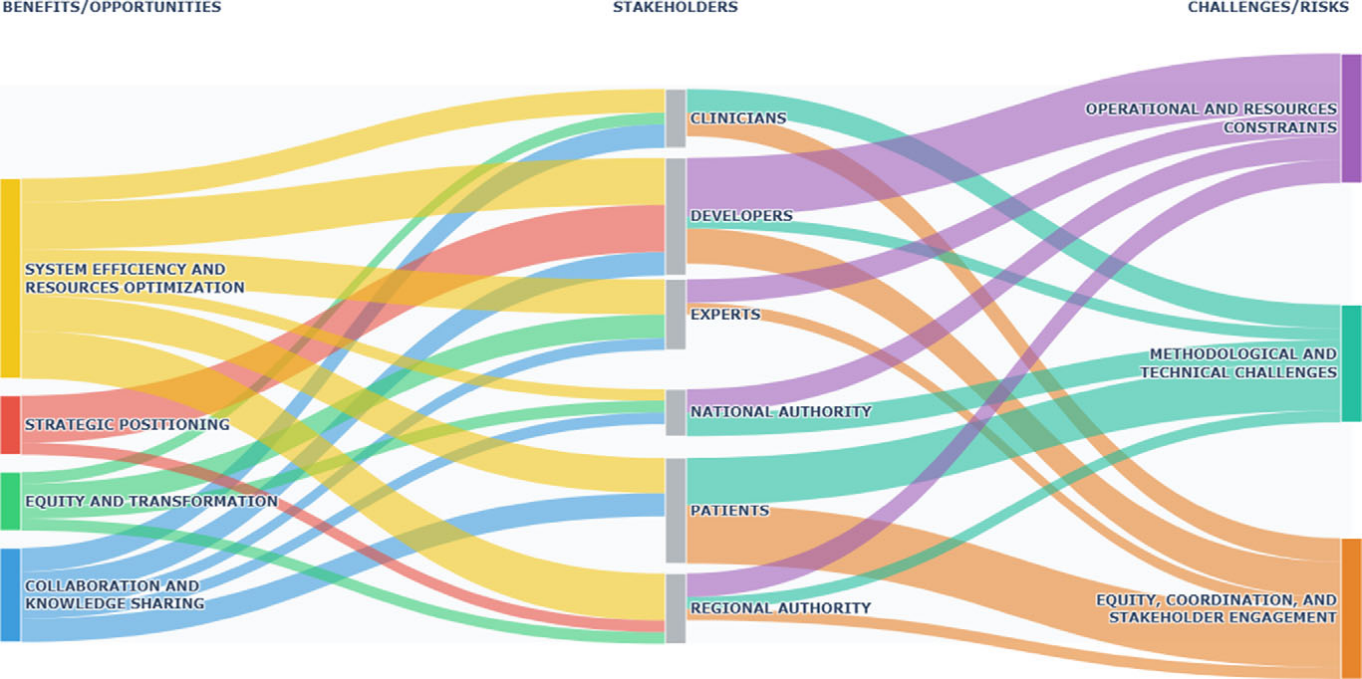
Expected benefits/opportunities and challenges/risks arising from the EU HTAR implementation according to different stakeholder group perspectives. *Note:* Based on the textual analysis of stakeholder interviews, individual quotations were clustered into broader categories of benefits and opportunities (left) and challenges and risks (right). The Sankey diagram displays, in a single visualization, what each stakeholder group (center) perceives as benefits/opportunities and challenges/risks. The thickness of each flow reflects the number of citations extracted for each stakeholder group. Further details on the clustering are reported in Supplementary Materials (Tables S2-S3).

**Figure 3.**
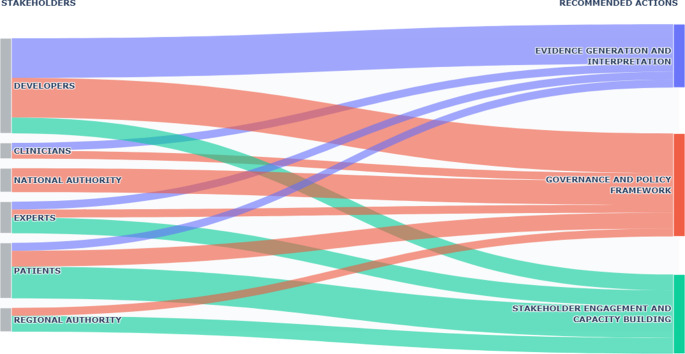
Recommended actions required to effectively implement the EU HTAR according to different stakeholder group perspectives. *Note:* Based on the textual analysis of stakeholder interviews, individual quotations were clustered into three overarching categories of recommended actions (right). The Sankey diagram provides a single visualization of how each stakeholder group (left) contributes to these action areas. The thickness of each flow reflects the number of citations extracted for each stakeholder group. Further details on the clustering are reported in Supplementary materials (Table S4).

Based on the interview topic guide, results were categorized into three broad categories, as shown in Figures 2 and 3 and summarized in the text below. A more detailed description, including illustrative quotas, is provided in Supplementary Material (S3-S5).

## Expected benefits and opportunities arising from the implementation of the HTAR

The numerous benefits reported by participants were clustered into four main topics: system efficiency and resources optimization; strategic positioning; equity and transformation; collaboration and knowledge sharing (Figure 2). The JCA, by avoiding duplication of assessments, allows different groups (i.e., national and regional authorities, and HTDs) to save time, reduce inefficiencies, and identify evaluation priorities, such as medicines yielding significant survival gains. Inputs provided to HTDs through the JSC can enhance the quality of clinical studies. The positive effects extend to the quality of clinical assessments, which is now progressively performed at an early stage and by experts recruited at a European level. The advancement and centralization of clinical assessment prevent it from becoming instrumental in financial and reimbursement decisions. The simplification and harmonization of procedures, as well as a greater integration across institutional levels, should reduce reimbursement decision times, which are currently long in Italy, and accelerate access to care. More uniform assessments through the JCA should reduce inter- and intra-country inequalities in patient access to innovative technologies. The JCA reports also support regional activities, including the identification of prescribing centers and the definition of diagnostic-therapeutic pathways.

The HTAR was perceived by national and regional HTA bodies, clinicians, and experts as an epochal change, comparable to the introduction of the EMA centralized procedure for marketing authorization some decades ago. The Regulation provides MS with the opportunity to share data, experiences, and resources, for the first time not on a voluntary basis but through an obligation, and generate and spread HTA knowledge both inside and outside the EU. The interest aroused at the international level around the HTAR should facilitate European countries and HTDs in attracting more investments in R&D and generating new research opportunities. Some benefits, such as efficiency and resources optimization (e.g., simplification of procedures, reduction of access time, avoidance of double or instrumental assessment), are transversal to multiple stakeholder groups, while others were more perceived by some stakeholder types. For example, the national HTA authority highlighted the importance of sharing knowledge and experience with other MS and also with non-EU countries. HTDs are particularly sensitive to the topic of strategic positioning, as they feel that the ‘dual’ role of AIFA, as both regulatory and HTA authority, can be further enhanced by the implementation of the HTAR, and also regional HTA bodies can benefit from high-quality, joint assessment reports. Clinical experts prioritize the quality of evidence generation, the time of patient access to medicines, and new data and research opportunities deriving from the EU.

## Expected risks arising from the implementation of the HTAR

The implementation of the HTAR also poses significant challenges, which were classified into three main topics: operational and resource constraints; methodological and technical challenges; equity, coordination, and stakeholder engagement (Figure 2). One of the main challenges is the extra effort that is required by both regulatory agencies and HTDs, and that is difficult to predict, being related to the number of new products that are subject to the JCA, which started in 2025 with anti-cancer medicines and ATMPs. A further but related issue is about the stringent deadlines required in the scoping phase for regulatory bodies involved in the PICO survey and for HTDs preparing the JCA dossier based on PICO data requirements. The number of PICOs to be considered by HTDs is also expected to be high even after consolidation, at a time when the available evidence might not cover all the comparators or the sub-populations included in the PICO framework. A major concern is the difficulty of generalizing a clinical assessment carried out at the European level to individual countries, which differ in terms of PICO requirements and are very likely to request additional assessments. The main challenge in this sense is the choice of comparator(s), especially for highly innovative cancer medicines or orphan drugs for which a standard of care may not exist in Europe, and trials are often single-arm. The duplication of assessments at the national level and the consequent double workload for HTDs might hamper the harmonization efforts and extend decision-making times for P&R. The HTD representatives were concerned about the management of different categories of products progressively falling under the scope of the HTAR. The first anti-cancer medicines that are subject to the JCA starting from 2025 could not benefit from the JSC tool under the HTAR during their development process (although they may have benefited from early dialogue or scientific advice from European HTA bodies under EUnetHTA Joint Action 3 and from parallel EMA-EUnetHTA JSC under EUnetHTA 21, until September 2023), but could have a ‘preferential lane’ in the national HTA process compared to medicines still referring to the national legislation only. The joint assessment of medical devices, which will start in 2026, is expected to be much more complex due to the limited amount of evidence available at the time of the JCA. HTDs and patient representatives complained about some inclusion criteria for joining the SN and the exclusion of smaller associations without a European representation. For example, patient associations in Italy may struggle to report their engagement in HTA development, given their limited involvement so far.

Some challenges, and mainly intercountry heterogeneity in evidence standards and the consequent risk of duplication of assessment, were perceived by stakeholders belonging to different groups, while others were specific to a particular group, such as the inequalities in access to JSC for medicines developed at different times, which is a concern of HTDs, or the still limited consideration of patient’s perspective reported by patients representatives. National and regional HTA bodies were mainly concerned about the readiness of MS and the availability of resources to comply with the EU procedures.

## Recommended actions

The participants interviewed recommended several actions to facilitate the progressive implementation of the HTAR at different levels. Based on their perceived benefits and risks, stakeholder recommendations were classified into three themes: evidence generation and interpretation; governance and policy framework; stakeholder engagement and capacity building (Figure 3). Despite conveying different concepts, the themes identified are not completely mutually exclusive (e.g., the promotion of inter-country collaborations is a key factor in harmonizing evidence standards for the JCA). HTDs should redefine companies’ priorities and global–local interactions, and “move earlier,” during the clinical trial design, to anticipate PICO data requirements, to schedule the data collection accordingly, and, especially, to identify a comparator representing the standard of care in most European countries. The harmonization of evidence standards across MS is key to facilitating this task. HTDs and patient representatives also expressed the need to adopt more flexible approaches for evidence generation (e.g., non-randomized studies, single-arm studies, indirect comparisons), especially in rare diseases and pediatric populations.

In terms of governance, to avoid duplication and conflicts among different authorities, institutional roles should be clearly redefined, and national and regional procedures adapted to the joint procedures (e.g., remodulating early access schemes to the JCA timelines). The political willingness of the EC to enforce the HTAR rules across the continent, and the individual countries’ ability to embrace a European perspective, alongside a widespread diffusion of the HTA culture, are key factors for the successful implementation of the HTAR. Regular updates of the HTAR are also desirable to adapt to the evolving HTA landscape in Europe.

Several participants reported that early dialogue and strategic collaboration should be incentivized among MS and all stakeholder types to obtain a uniform application of the EU rules. According to AIFA and patient representatives, relaxing the inclusion criteria and other participation rules in the SN is essential to foster collaboration with different types of stakeholders. All types of stakeholders agreed that higher investment in education and training is required at different levels to comply with the EU processes, by creating new professional figures and upgrading the existing skills. Patient education should be enhanced with public financing to avoid conflicts of interest in the SN and address the whole HTA process.

Some initiatives are supported by multiple stakeholders and include the adaptation of national and regional procedures to the European processes, the promotion of strategic collaboration across different actors, and education and training in HTA. In addition, representatives of HTDs expressed the need for redefining priorities and roles within their organizations, designing clinical studies that respond to both regulatory and HTA requirements, and developing beyond trial-based approaches to evidence generation (e.g., network meta-analysis). Clinicians mainly recommended the harmonization of evidence standards, and patient representatives a broader involvement of patients in HTA processes, following the example of the National Institute for Health and Care Excellence (NICE) in the UK.

## Discussion

This study aimed to investigate the potential impact of the HTAR from the perspective of multiple stakeholders. Drawing on a literature review and qualitative analysis of in-depth stakeholder interviews, we evaluated the potential implications of the HTAR at the national level and organized them around broader topics, using Italy as an in-depth case study. The HTAR is a decisive milestone on the path toward a more coordinated and sustainable HTA cooperation across Europe (32). The establishment of a clear set of UE rules is expected to increase the speed, consistency, methodological rigor, and predictability of clinical assessment, with larger benefits for MS with limited HTA expertise. This should reduce duplication of effort and administrative burden, gain efficiency, streamline decision-making, and ultimately foster more timely and equitable access to innovative health technologies throughout the EU (5;33).

However, the effective implementation of JCAs across diverse national contexts remains a complex challenge. The HTAR mandates MS to take JCA reports into account at the national level, but questions remain regarding the harmonization of the JCA implementation. The observed variations in PICO criteria across the national HTA bodies highlight the difficulties in achieving a unified approach, already during the JCA scoping phase (34). Moreover, the inefficient utilization of JCA reports at the national level, due to their inadequate consideration with the resulting risk of duplication and overlapping of processes, could extend national decision-making and hinder, rather than facilitate, patient access (35). On the HTD’s side, the main complexity is dealing with multiple PICOs after consolidation and with a tight timeframe for preparing the JCA dossier, which might prolong the time to market of health innovations (4). In infectious diseases, heterogeneity in the available data and differences in national vaccine calendars may lead to an even higher number of PICO per submission (36).

The perspectives gathered in this study highlight both the potential and the challenges of applying EU-level assessments within national regulatory and reimbursement systems, especially in a decentralized system such as the Italian one. While JCA is expected to significantly improve evaluation quality and efficiency, its successful integration will depend on the ability of individual countries to adapt governance structures, align evidence requirements, and coordinate institutional processes at different levels without undermining the intended harmonization. The EC can facilitate this process by executing a thorough communication strategy to eliminate countries’ uncertainty around implementation, engage different healthcare system stakeholders, and secure commitment from all 27 MS to collaborate in the JCA process (33). The process of harmonization, however, must be implemented while respecting each country’s autonomy in pricing, reimbursement, and budget management, which remain under national jurisdiction.

In Italy, the recent reform of the national HTA and regulatory body (i.e., AIFA) may favor the alignment of the national procedures with the EU ones (37;38). The national HTA and P&R procedure, now managed by the single Scientific and Economic Committee, promotes the early evaluation of medicinal products subject to the JCA. Moreover, AIFA has already developed expertise in line with the HTAR, such as objective criteria for evaluating the added therapeutic value and the use of GRADE for assessing the quality of evidence within the attribution of therapeutic innovativeness (38;39). In April 2025, AIFA established the Working Group on the HTAR, which takes part in the joint assessment of medicines and in the JSC procedures according to the EU legislation (40). Moreover, AIFA is part of the Heads of HTA Agencies Group (HAG), a high-level forum aiming to strengthen and support the use of HTA in Europe, including the implementation of the HTAR (38). Although harmonization is beneficial for ensuring faster access to new health technologies and avoiding redundant efforts in the evaluation of clinical evidence, the Agency emphasizes the importance of maintaining national autonomy in assessing non-clinical aspects of health technologies (e.g., cost-effectiveness, ethical issues), and of adapting JCA outcomes to the national healthcare priorities. (41). The establishment of a national network might be useful to develop a shared PICO across different institutional levels, contributing to the development of a JCA that is more aligned with country-specific health needs and better integrated into AIFA’s HTA and P&R procedures (42).

Therefore, across Europe, it is imperative to foster active stakeholder engagement, invest in capacity building – including HTA literacy and methodological training, especially for patients – and promote flexible yet coherent implementation strategies. A better inter-country and intra-country alignment with respect to clinical HTA standards, along with skillful anticipation of the right PICOs by manufacturers, should be promoted. From the clinicians’ perspective, the provisions of guidelines on the quality standards of evidence, clinical trial design, comparator(s), and endpoints at the EU level may serve this purpose. In this way, the MS could take full advantage of the benefits that the JCA can offer by integrating and completing the capabilities of individual national processes (20). The transitional phase provides not only a regulatory challenge but also a unique opportunity to redefine the role of HTA in supporting equitable, evidence-based, and value-driven healthcare systems across Europe.

The study contributes to the still-limited body of scientific literature around the implementation of the HTAR at the EU country level. The results obtained for the Italian context are aligned with previous findings from semi-structured interviews with representatives of national and European institutions, ATMP developers, sickness funds, and academics in Belgium. They revealed that the main challenges are compiling a harmonized PICO, adapting local procedures, and increasing capacity to actively participate in the JCA and JSC. National/regional HTA bodies and payers must act to adopt the EU procedures within their national legislation, invest in training and education, revise their timelines, and prepare for interactions at an EU level (5). Similarly, the major implications of the HTAR for the Dutch HTA authority are that the scoping phase begins much earlier and the JCA report is the starting point for the national assessment. The Dutch National Health Care Institute (ZIN) prepared for this by performing a continuous gap analysis of the current assessment process (43).

However, this study has some limitations. Although we recruited participants from six different stakeholder groups and tried to reach content saturation with more than one interview per stakeholder group (except for the national authority), it is possible that we missed some relevant insights. Moreover, the literature review ended in December 2024, when all interviews were completed, but the HTAR was still not implemented, and responses were based on interviewees’ experience, opinions, and expectations. Therefore, the 16 included studies might have highlighted opportunities that did not arise and barriers that have already been resolved during the first implementation phase. Future research will consider updating the literature review to focus on the actual changes the JCA of selected health technologies and other HTAR-related activities have brought about for individual countries during their first year of application.

## Supporting information

10.1017/S026646232610364X.sm001 Meregaglia et al. supplementary material Meregaglia et al. supplementary material

## Supplementary material

The supplementary material for this article can be found at http://doi.org/10.1017/S026646232610364X.
