# Supplementary material for: Implementing the EU HTA regulation and joint clinical assessment: a multi-stakeholder perspective from Italy
Source: Int J Technol Assess Health Care. 2026 Apr 13;42(1):e39. doi: 10.1017/S026646232610364X (PMC13078104; doi:10.1017/S026646232610364X)
Supplement: Meregaglia et al. supplementary material [file S026646232610364Xsup001.zip › Supplementary file S2.docx]

**Supplementary File 2.**

**Table S1.** Interviews.

| Interview code | Category | Time | Duration |
| --- | --- | --- | --- |
| N1 | National HTA authority | November 2024 | 26:52 |
| R1 | Regional HTA authority | December 2024 | 22:03 |
| R2 | Regional HTA authority | November 2024 | 25:58 |
| D1 | Health technology developers | December 2024 | 37:39 |
| D2 | Health technology developers | December 2024 | 24:53 |
| D3 | Health technology developers | December 2024 | 19:27 |
| C1 | Clinicians | November 2024 | 20:41 |
| C2 | Clinicians | November 2024 | 6:38 |
| P1 | Patient representatives | November 2024 | 42:24 |
| P2 | Patient representatives | November 2024 | 16:29 |
| P3 | Patient representatives | November 2024 | 25:14 |
| E1 | Experts | November 2024 | 37:09 |
| E2 | Experts | December 2024 | 21:02 |
