# Supplementary material for: Implementing the EU HTA regulation and joint clinical assessment: a multi-stakeholder perspective from Italy
Source: Int J Technol Assess Health Care. 2026 Apr 13;42(1):e39. doi: 10.1017/S026646232610364X (PMC13078104; doi:10.1017/S026646232610364X)
Supplement: Meregaglia et al. supplementary material [file S026646232610364Xsup001.zip › Supplementary file S3.docx]

**Table S2.** Expected benefits and opportunities arising from the HTAR implementation according to different stakeholder group perspectives.

| **Benefit / Opportunity** | **Illustrative quotes** |
| --- | --- |
| **SYSTEM EFFICIENCY AND RESOURCES OPTIMIZATION** | |
| Simplification of procedures and shorter approval and access times | “The availability of comprehensive scientific reports at European level will speed up national assessment for P&R” (N1) |
|  | “The regulation should lead to greater integration between regulatory bodies, with the aim of simplifying procedures and reducing access times to health technologies” (R1) |
|  | “The joint evaluations will facilitate faster access to medicines” (R2) |
|  | “Currently, approval and reimbursement times are still long: on average 14 months for national reimbursement, plus another 10 months for regional access. The largest benefit I see is precisely that of accelerating these times, as all evaluation is done at European level” (D1) |
|  | “Currently, the times are very slow, years pass from the moment a study is completed to when the drug is actually available in clinical practice. Therefore, a simplification and speeding up of procedures is welcome” (C1) |
|  | “The HTAR should guarantee a faster path and early access to new therapies; for some patients a few days can make a difference” (P2) |
|  | “The spirit of the HTAR and the JCA is exceptional, because the aim is to accelerate access to the drug, increase the homogeneity of procedures and improve transparency” (P3) |
| Identifying priorities, efficient use of public resources | “Sometimes clinicians and patients push for the adoption of drugs or devices that are useless according to the available evidence. An external independent evaluation, based on scientific and objective parameters, can help educate professionals and patients, avoiding waste of public resources”. “The JCA may also highlight cases where rapid access is not necessary, avoiding the mantra of ‘access at all costs’” (R2) |
|  | “As a major advantage, I expect the JCA will favour truly transformative medicines impacting important outcomes, such as survival” (C2) |
| Avoidance of double or instrumental assessment | “Regions should not make evaluations except in exceptional cases, because they have limited resources for HTA and this may slow down access to drugs and devices” (R1) |
|  | “The centralization of the assessment process means that is also temporally far from the time of price negotiations and avoids that scientific evaluations become instrumental to reimbursement and budget impact considerations” (D2) |
|  | “The JCA is a common assessment that, if accepted by individual States, allows for savings in resources, time and work.” “This new approach will change the procedures of manufacturing companies, which will be able to prepare a single documentation for all countries, instead of adapting it for each of the 27 MS” (E1) |
|  | “The methodology is clearly based on a single submission of data by HTDs at European level, which should reduce duplications and inefficiencies in the evaluation processes” (E2) |

**Table S2.** Expected benefits and opportunities arising from the HTAR implementation according to different stakeholder group perspectives.

| **SYSTEM EFFICIENCY AND RESOURCES OPTIMIZATION (cont.)** | |
| --- | --- |
| Increase in the quality of assessments | “The assessment performed at a higher institutional level, in an early phase and through the involvement of different stakeholders in a homogeneous, inclusive and well-organized process should lead to an increase in quality over time” (D2) |
|  | “Joint reports and sharing of methodologies, especially for indirect comparisons, can improve the quality of evaluations in Italy” (D3) |
|  | “I expect that the recruitment of experts will take place at a higher level, and this will be reflected in the quality of the evaluations” (P1) |
|  | “If the system works, the benefits will be significant: reduced times and potentially improved quality and costs, thanks to the aggregation of skills at a central level” (E1) |
| **STRATEGIC POSITIONING** | |
| Investment attraction in Europe, generation of a single market | “Europe can become a competitive hub for research and development, attracting investment in both production and innovation” (D1) |
|  | “Gradually, assessment elements (e.g., comparators) and procedure will tend to be similar across MS. This convergence process will attract investments in R&D and generate, in a decade, a single EU market” (D2) |
| Enhancement of AIFA’s strategic role | “AIFA is the only agency in Europe to have a dual role, both regulatory and HTA. This means that AIFA representatives sit both in the EMA and in JCA and JSC groups” (D1) |
| Support to regional decisions (e.g., identification of prescribing centres) | “A joint European evaluation can also benefit the local level, facilitating the definition of prescribing centres and the adoption of regional therapeutic plans. For example, we can decide whether to supply drugs through direct or affiliated distribution, and favour those with a better cost-effectiveness ratio” (R2) |
|  | “Regional authorities will access a joint assessment report on the relative efficacy and safety, which can guide organizational, ethical and social evaluations. This will allow to identify prescribing centres earlier, create or modify diagnostic-therapeutic pathways and, in general, ensure faster access to therapies” (D1) |
| **EQUITY AND TRANSFORMATION** | |
| Epochal change, great opportunity | “The HTAR represents a milestone in the history of the pharmaceutical sector. This change can be compared to the establishment of the EMA and the introduction of centralized procedures for the authorization of medicines. Some effects will occur in the short term, while others will emerge in the medium-long term” (N1) |
|  | “The new regulation is comparable to the establishment of the EMA in the 90s” (E1) |
| More equitable access across countries and within countries | “The HTAR could allow for more uniform evaluation, promoting greater equity, not only between countries, but also at a national level” (R2) |
|  | “There are currently disparities in access at European level, that could be reduced by the new HTAR” (C1) |
|  | “The HTAR can reduce regional inequalities in Italy, because the decisions would be based on criteria finally shared at European level” (E2) |

**Table S2.** Expected benefits and opportunities arising from the HTAR implementation according to different stakeholder group perspectives.

| **COLLABORATION AND KNOWLEDGE SHARING** | |
| --- | --- |
| Recognition of patient’s role in HTA | “For the first time, the patient is fully recognized as stakeholder in HTA” (P2) |
| More research project opportunities | “The availability of new data will allow for more in-depth statistical analyses (e.g., can the ESMO score predict the outcome of the JCA and the timing of approval?)” (C1) |
| Inputs from JSC to design clinical trials | “This consultation allows companies to interface with authorities and obtain crucial input for the design of phase three clinical studies” (D1) |
|  | “The topics we talk about are very complex and discussion helps a lot, making both parties responsible” (D2) |
|  | “Clinicians often wonder whether study design could be improved, so they welcome the opportunity given to companies to seek advice from HTA agencies (C1) |
| Sharing data, experiences, knowledge, and resources with other countries | “For the first time, not only in a cooperative and voluntary manner, but through an obligation, 27 UE MS will sit at the same table, cooperate, share methodologies, orientations and also practical work”  “Instead of publications from single national agencies, there will be a shared JCA report in English on the EC website. This will contribute to spreading knowledge among the EU countries and outside” (N1) |
|  | “As patients, we have the opportunity to learn from other countries and bring our concerns to the European level. We are no longer a drop in the ocean; we are the ocean” (P1) |
|  | “The centralization will be particularly useful for small MS without their own HTA authority and limited HTA expertise and procedures” (E1) |

AIFA: Italian Medicines Agency; EMA: European Medicines Agency; ESMO: European Society of Medical Oncology; HTA: health technology assessment; HTAR: health technology assessment regulation; HTDs: health technology developers; JSC: joint scientific consultation; MS: Member State; R&D: Research and Development.

C: clinicians; D: developers; E: experts; N: national authority; P: patients ; R: regional authority
