# Supplementary material for: Implementing the EU HTA regulation and joint clinical assessment: a multi-stakeholder perspective from Italy
Source: Int J Technol Assess Health Care. 2026 Apr 13;42(1):e39. doi: 10.1017/S026646232610364X (PMC13078104; doi:10.1017/S026646232610364X)
Supplement: Meregaglia et al. supplementary material [file S026646232610364Xsup001.zip › Supplementary file S4.docx]

**Table S3.** Expected challenges and risks arising from the HTAR implementation according to different stakeholder group perspectives.

| **Challenge / Risk** | **Illustrative quotes** |
| --- | --- |
| **OPERATIONAL AND RESOURCES CONSTRAINTS** | |
| Excessive workloads and lack of human resources, unpreparedness of countries | “An issue is the lack of human resources in all MS, including the most prepared. Workloads will be intense and difficult to predict, e.g., at least 20 medicines are expected to be subject to JCA in 2025” (N1) |
|  | “The only risk is that not all MS are ready to start with the new procedures and aware of the importance of the regulatory change underway” (R2) |
|  | “In the first three years, there will be around 80 drugs to be evaluated: 17 oncology drugs and 8 advanced therapies in the first year alone” “I hope that AIFA receives the necessary resources - both in terms of personnel and skills - to deal with the workload required by the new regulation” (D1) |
| Stringent deadlines for PICO requirements and JCA dossier | “There are critical issues such as stringent deadlines: some phases, e.g., the PICO survey, require very short times, which could overload the system” (N1) |
|  | “To prepare the JCA dossier, we have a very limited period of time: 100 days, and only 60 for accelerated procedures” (D1) |
|  | “AIFA will have a role in the scoping process, but the timing of the CSE meetings may not align with those of the PICO survey”  “To satisfy PICO data requirements, companies must work on the molecule 18 months before submission to the EMA and 3-4 years before the launch in Italy, a timeline that, to date, we are unready to meet”.  “When the final PICOs arrives, the time to respond is really short (2-3 weeks)” (D3) |
| Too many PICO to be considered | “Having many PICOs to consider means that companies will have to produce a lot of evidence, with the risk that some populations may not be covered” (D1) |
|  | “The quantity of PICOs that will be produced is relatively high, therefore the process will be fairly energy-intensive for companies” (D2) |
|  | “A crucial issue is how feedback on PICOs from individual MS will be handled during the scoping phase (e.g. does the opinion of a large country count as much as that of a small country?)” (E1) |
| More complex evaluation of medical devices | “The evaluation of devices is much more complex than that of drugs, due to the lesser available literature and limited evidence” (R2) |
|  | “The centralized technology assessment will address also selected medical devices and other technologies, with greater complexity than pharmaceuticals” (E1) |

**Table S3.** Expected challenges and risks arising from the HTAR implementation according to different stakeholder group perspectives.

| **METHODOLOGICAL AND TECHNICAL CHALLENGES** | |
| --- | --- |
| Lack of independent analyses for the JCA | “At present, the assessments are based exclusively on the data provided by HTDs, without any independent analyses. This could lead companies to omit the least favourable comparisons.” (N1) |
| Identification and limitations of standard comparator | “Comparator is the most critical element. Except for advanced therapies or absolutely innovative drugs, finding a standard one is a problem. Many studies use non-standard comparators or single arms” (R2) |
|  | “I fear that we will continue to do joint assessments on registration studies that often have a “suboptimal” control arm (e.g., an “old” standard of care)”.  “Clinicians often rely only on network meta-analyses to choose between alternatives, due to the lack of direct comparisons” (C1) |
|  | “The comparator drug may not be the most up-to-date in some countries” (C2) |
| Too restrictive inclusion criteria and limited representativeness in the SN | “The SN only includes associations with European representation, excluding some associations that represent relevant medical conditions or medicinal products (e.g., vaccines)” (D1) |
|  | “Only the largest associations, with greater visibility at European level, are included in the network”.  “We have to find a balance between transparency and pragmatism. For example, asking a clinician not to have interacted with HTDs, or a patient to have been involved in HTA processes, is unrealistic” (P2) |
| Data confidentiality and COI management in the SN | “The HTAR is very inclusive and involve all stakeholders. However, relevant issues related to data confidentiality emerge, e.g., how to handle commercially sensitive items, or how to identify clinical experts and patients completely free from COI” (N1) |
|  | “Patient education is funded by HTDs, and both parties are included in the SN” (P1) |
|  | “Highly experienced patients may have had interactions with pharmaceutical companies and be excluded from the network, thus losing their relevant contribution” (P2) |
|  | “It is relatively easy to verify the existence of COI for an association through balance sheets. Conversely, individual experts cannot be asked to provide a tax return or bank statement for privacy reasons” (P3) |
| **EQUITY, COORDINATION, AND STAKEHOLDER ENGAGEMENT** | |
| Limited consideration of patient’s perspective | “PROMs and PREMs are fundamental instruments to assess the impact of a new technology, but they are not adequately considered by the HTAR” (P1) |
|  | “The patient can provide useful information on living with the condition, but the risk is that their contribution remains only symbolic” (P2) |
|  | “It is important to understand how patient involvement will be used. We would like to see patient’s opinions actually taken into consideration” (P3) |

**Table S3.** Expected challenges and risks arising from the HTAR implementation according to different stakeholder group perspectives.

| **EQUITY, COORDINATION, AND STAKEHOLDER ENGAGEMENT (cont.)** | |
| --- | --- |
| Inequalities in clinical assessment and access to scientific consultation | “The first medicines to undergo the JCA (i.e., cancer drugs and advanced therapies) could not benefit from this tool [the JSC]. Moreover, the slots available for a JSC are very few: there are only 5 or 7 for 2025” (D1) |
|  | “The risk is that products falling within the European regulation from 2025 might have a preferential lane compared to others” (D3) |
| Intercountry heterogeneity, duplication of assessment, lengthening of decisions. | “There is the risk of duplication of assessments, with a consequent lengthening of decision time, and the emergence of conflicts across MS, due to differences in national health systems and access rules” (R1) |
|  | “Without a sound and participated JCA dossier, each national agency, including AIFA, could repeat its own assessment process and request new data from companies, thus hampering harmonization efforts” (D1) |
|  | “I am not sure that decision-making times will actually be reduced, since the HTAR leaves wide discretion to MS that can request complementary analyses on aspects not considered in the JCA” (C1) |
|  | “There is a risk of delay in negotiating the price and in drug access, especially for drugs providing marginal benefits” (C2) |
|  | “In case of uncertainties or difficulties in generalizing JCA results emerging at European level, MS could request more complementary assessments and extend decision-making times”.  “I see the difficulty in contextualizing at national level assessments carried out at European level”.  “A randomized study conducted in one country may not be representative of the entire European population” (P1) |
|  | “However, decision times for price and reimbursement may remain unchanged” (P2) |
|  | “Finding a shared methodology, accepted by all MS, minimizing variability and defining common criteria is a challenge, especially considering current differences in terms of study design and endpoints”.  “It depends on how much this change will be accepted, especially by countries with larger HTA agencies. In case of doubt, some could request additional data, resulting in double work for HTDs” (E1) |

COI: conflict of interest; EMA: European Medicines Agency; HTDs: health technology developers; JCA: Joint Clinical Assessment; JSC: Joint Scientific Consultations; MS: Member State; PICO: population, intervention, comparator, outcome. PREM: patient-reported experience measure; PROM: patient-reported outcome measure.
