# Supplementary material for: Implementing the EU HTA regulation and joint clinical assessment: a multi-stakeholder perspective from Italy
Source: Int J Technol Assess Health Care. 2026 Apr 13;42(1):e39. doi: 10.1017/S026646232610364X (PMC13078104; doi:10.1017/S026646232610364X)
Supplement: Meregaglia et al. supplementary material [file S026646232610364Xsup001.zip › Supplementary file S5.docx]

**Table S4.** Actions required to effectively implement the HTAR according to different stakeholder group perspectives.

| **Actions required** | **Illustrative quotes** |
| --- | --- |
| **EVIDENCE GENERATION AND INTERPRETATION** | |
| Preparatory work, moving early in R&D | “It is essential to start much earlier, already in the design phase of phase III clinical studies, to identify the best comparator, i.e. the one that represents the standard for the majority of European countries.” “Until now, registration studies were mainly designed from a regulatory perspective, focusing on the benefit-risk profile. With the HTAR, the pivotal study must respond to both regulatory and HTA requirements, including data on comparative efficacy and safety, which are useful for market access” (D1) |
|  | “There will be a lot of preparatory work [for companies] to define PICOs and to support both medical division and market access in the initial stages” (D2) |
| New and flexible approaches to evidence generation | “In some cases, it is not possible to include a certain comparator in the phase III clinical studies, and additional evidence generation is required to collect data in missing subpopulations or comparators” (D1) |
|  | “It is necessary to increase knowledge on evidence generation processes, which is scarce in many companies, e.g., the market access is familiar with non-randomized studies, while the medical division much less” (D2) |
|  | “Even for drugs for which the HTAR will only apply from 2030 (e.g., in neurology), companies should start using public methodologies (i.e., indirect comparisons) to improve the quality of evaluations” (D3) |
|  | “In some cases, such as for emerging technologies and rare and paediatric diseases, randomized clinical studies are unfeasible. The JCA should also consider evidence from single-arm studies as valid” (P3) |
| Harmonization of evidence standards | “It is important to avoid that the comparators chosen at European level are suboptimal for some MS” (C2) |
|  | “Over the next few months or years, there will be significant work to harmonize the evaluation standards” (E1) |
| **GOVERNANCE AND POLICY FRAMEWORK** | |
| Regular updates of the HTAR | “The regulation will be reviewed after two years, which could lead to the introduction of corrective measures” (N1) |
| Redefinition and adaptation of national and regional procedures | “To avoid duplications and conflicts, it is essential to clearly define rules, roles and times at each institutional level”. “Regional Commissions should understand and implement the JCA reports” (R1) |
|  | “The CSE will support AIFA with a mandatory, but non-binding, opinion on the PICO criteria, which may be modified during the consolidation phase” (N1) |
|  | “For example, in Italy and other countries, for some innovative drugs the dossier can be submitted immediately after receiving a positive opinion from the EMA. With the HTAR, the JCA report will only be available 30 days after the EMA decision, creating a time gap that will have to be managed” (D1) |
|  | “It will be important to understand how, at MS level, it is possible to re-modulate the early access modalities with respect to the new JCA timelines” (C1) |
|  | “The regional authorities will have to integrate these assessments into the decision-making processes and paths, also considering the local heterogeneity and population needs” (E2) |

**Table S4.** Actions required to effectively implement the HTAR according to different stakeholder group perspectives.

| **GOVERNANCE AND POLICY FRAMEWORK (cont.)** | |
| --- | --- |
| Redefinition of company’s priorities and roles for HTDs | “The further in time we are from the product launch, the more processes are delegated to the global level. We are preparing for this, and I believe all companies are preparing for this” (D2) |
|  | “It was initially thought that centralized scientific assessment would reduce pressure on individual countries. Instead, more support is required also at local level, and global-local interactions have to be redefined”.  “The new process will require a review of skills, priorities and internal organization, especially for national branches (or subsidiaries), which are mainly commercial and not involved in R&D” (D3) |
| Relaxing the rules of the SN | “A change in the eligibility criteria and other rules of the SN will be required to facilitate the interaction with relevant stakeholders” (N1) |
|  | “In the SN, patient associations represent the interests of their members, while in the UK, NICE involves also individual patients in the HTA process” (P1) |
|  | “The solution might be to openly declare all interests but be less rigid in involving patients and do not exclude a priori just because of the relationships with companies” (P2) |
| Embracing a European perspective | “We need to get out of our comfort zone and embrace a European perspective” (D1) |
|  | “A key factor will be Europe's political intent and ability to enforce the new rules. However, if countries do not adhere to this political vision, to maintain their autonomy or for reasons related to differences in spending or regulations, the whole process risks failing” (D2) |
| **STAKEHOLDER ENGAGEMENT AND CAPACITY BUILDING** | |
| Promotion of strategic collaborations | “Strategic collaboration between healthcare institutions, patients/citizens and pharmaceutical/medical device companies should be promoted” (R1) |
|  | “It is crucial to collaborate with patient groups, scientific communities and local agencies to understand unmet treatment needs” (D1) |
|  | “Dialogue with associations from other countries is always beneficial, to find answers to shared problems” (P2) |
|  | “Greater collaboration among Regions is essential to apply the new assessment criteria consistently throughout Italy” (E2) |
| Education and training needs, diffusion of HTA culture | “Adequate training, upgrade of skills and review of working methods are required for all stakeholders” (R2) |
|  | “The introduction of new professional figures and an intense training activity have been required, which involved not only the market access team but also the regulatory and medical teams”. “A greater HTA culture diffusion is needed, involving academia, patient associations, scientific societies, clinicians and companies” (D1) |
|  | “It is essential to invest in training and resources (e.g., IT infrastructure) to address the new requirements and receive the new documentation, also at regional level” (E2) |

**Table S4.** Actions required to effectively implement the HTAR according to different stakeholder group perspectives.

| **STAKEHOLDER ENGAGEMENT AND CAPACITY BUILDING (cont.)** | |
| --- | --- |
| Enhancing patient education and involvement | “Patient education must be publicly funded to avoid conflicts of interest in the SN” (P1) |
|  | “Patient education should address the entire HTA process, not just pharmacoeconomics” (P2) |
|  | “We are working on the creation of a “Summary of Information for Patients” (SIP) written in an accessible language, which should be included in the JCA dossier submitted by companies. This practice has already been tested in Australia and in the United Kingdom, and we hope that could be pilot tested by the European CG” (P3) |

CG: coordination group: MS: Member State; HTAR: Health Technology Assessment Regulation; HTDs: health technology developers; JCA: joint clinical assessment; NICE: National Institute for Health and Care Excellence; R&D: research and development; SN: stakeholder network.

C: clinicians; D: developers; E: experts; N: national authority; P: patients; R: regional authority
