# Supplementary material for: Implementing the EU HTA regulation and joint clinical assessment: a multi-stakeholder perspective from Italy
Source: Int J Technol Assess Health Care. 2026 Apr 13;42(1):e39. doi: 10.1017/S026646232610364X (PMC13078104; doi:10.1017/S026646232610364X)
Supplement: Meregaglia et al. supplementary material [file S026646232610364Xsup001.zip › Supplementary file S1.docx]

**File S1.** Interview guide.

This interview focused on the following questions:

1. *Based on your experience, to what extent will the activities envisaged by the regulation (in particular, the joint clinical evaluation, JCA) impact on the processes and operating methods of your organization or professional category and, more generally, the development of new healthcare technologies, HTA and P&R determination procedures, and access to medicines at the national/regional level?*
2. *What potential benefits or advantages do you foresee from implementing the new regulation?*
3. *At the same time, do you believe the new regulation could also pose risks or critical issues? If so, what are they?*
4. *In conclusion, in your opinion, what changes will be necessary within your organization or professional category, and more generally within the country system, to address the new activities and, in particular, the JCA of new oncology drugs and advanced therapies starting in 2025?*

* This translation from Italian is intended for publication purposes and might not represent a validated translation.
